# Epinephrine’s effects on cerebrovascular and systemic hemodynamics during cardiopulmonary resuscitation

**DOI:** 10.1186/s13054-020-03297-4

**Published:** 2020-09-29

**Authors:** Constantine D. Mavroudis, Tiffany S. Ko, Ryan W. Morgan, Lindsay E. Volk, William P. Landis, Benjamin Smood, Rui Xiao, Marco Hefti, Timothy W. Boorady, Alexandra Marquez, Michael Karlsson, Daniel J. Licht, Vinay M. Nadkarni, Robert A. Berg, Robert M. Sutton, Todd J. Kilbaugh

**Affiliations:** 1 Division of Cardiothoracic Surgery, The Children’s Hospital of Philadelphia, 3401 Civic Center Blvd, Philadelphia, PA 19104 USA; 2 Division of Cardiovascular Surgery, The University of Pennsylvania, Philadelphia, PA USA; 3 Department of Neurology, The Children’s Hospital of Philadelphia, Philadelphia, PA USA; 4 Department of Anesthesiology and Critical Care Medicine, The Children’s Hospital of Philadelphia, Philadelphia, PA USA; 5 Department of Pediatrics, Division of Biostatistics, Children’s Hospital of Philadelphia, Philadelphia, PA USA; 6 Department of Pathology, University of Iowa Carver College of Medicine, Iowa City, IA USA; 7 Department of Neurosurgery, Rigshospitalet, Copenhagen, Denmark

**Keywords:** Cardiopulmonary resuscitation, Cerebral blood flow, Cerebral blood flow and metabolism, Diffuse correlation spectroscopy, Diffuse optical spectroscopy

## Abstract

**Background:**

Despite controversies, epinephrine remains a mainstay of cardiopulmonary resuscitation (CPR). Recent animal studies have suggested that epinephrine may decrease cerebral blood flow (CBF) and cerebral oxygenation, possibly potentiating neurological injury during CPR. We investigated the cerebrovascular effects of intravenous epinephrine in a swine model of pediatric in-hospital cardiac arrest. The primary objectives of this study were to determine if (1) epinephrine doses have a significant acute effect on CBF and cerebral tissue oxygenation during CPR and (2) if the effect of each subsequent dose of epinephrine differs significantly from that of the first.

**Methods:**

One-month-old piglets (*n* = 20) underwent asphyxia for 7 min, ventricular fibrillation, and CPR for 10–20 min. Epinephrine (20 mcg/kg) was administered at 2, 6, 10, 14, and 18 min of CPR. Invasive (laser Doppler, brain tissue oxygen tension [PbtO_2_]) and noninvasive (diffuse correlation spectroscopy and diffuse optical spectroscopy) measurements of CBF and cerebral tissue oxygenation were simultaneously recorded. Effects of subsequent epinephrine doses were compared to the first.

**Results:**

With the first epinephrine dose during CPR, CBF and cerebral tissue oxygenation increased by > 10%, as measured by each of the invasive and noninvasive measures (*p* < 0.001). The effects of epinephrine on CBF and cerebral tissue oxygenation decreased with subsequent doses. By the fifth dose of epinephrine, there were no demonstrable increases in CBF of cerebral tissue oxygenation. Invasive and noninvasive CBF measurements were highly correlated during asphyxia (slope effect 1.3, *p* < 0.001) and CPR (slope effect 0.20, *p* < 0.001).

**Conclusions:**

This model suggests that epinephrine increases CBF and cerebral tissue oxygenation, but that effects wane following the third dose. Noninvasive measurements of neurological health parameters hold promise for developing and directing resuscitation strategies.

## Background

Epinephrine remains a mainstay in the treatment of cardiac arrest, but there continues to be debate on its safety and efficacy [1–5]. In various pediatric and adult studies of cardiac arrest, early epinephrine administration was associated with increased rates of return of spontaneous circulation (ROSC) and improved neurological outcomes when compared with late administration [6–9]. Conversely, there are several studies suggesting that epinephrine does not improve outcomes and may possibly confer a harmful effect [10–12]. In the large PARAMEDIC-2 randomized double-blind clinical trial of epinephrine versus placebo for out-of-hospital cardiac arrests in adults, the use of epinephrine improved the 30-day survival rate, but did not improve survival with favorable neurologic outcomes [3].

A fundamental issue with epinephrine’s mixed effects in cardiopulmonary resuscitation (CPR) lies in an incomplete understanding of its mechanistic effects on cerebrovascular hemodynamics. While epinephrine increases systemic arterial blood pressure, there are conflicting experimental results regarding its effect on cerebral blood flow (CBF) and cerebral tissue oxygenation. Specifically, animal studies have demonstrated both increases [13–17] and decreases [18–21] in various measurements of CBF following epinephrine administration during CPR. Maintaining CBF during CPR is an important physiologic target to minimize potentially devastating effects of cerebral hypoperfusion during this vulnerable period. Thus, better delineation of the effect of epinephrine on CBF and cerebral tissue oxygenation during CPR is an important knowledge gap in the quest to improve both mortality and neurologic morbidity following cardiac arrest.

In this study, we assessed the cerebrovascular hemodynamic and cerebral tissue oxygenation effects of intravenous bolus-dosed epinephrine, using both invasive and noninvasive measurements of CBF and cerebral oxygen saturation in a swine model of asphyxia-associated cardiac arrest. We hypothesized that (1) repeated epinephrine doses during CPR will increase CBF and cerebral tissue oxygenation, (2) the effects of repeated epinephrine administration are attenuated with subsequent doses, and (3) noninvasive measurements of CBF and cerebral oxygenation directly correlate with invasive measurements.

## Methods

All procedures were approved by the Institutional Animal Care and Use Committee at the Children’s Hospital of Philadelphia and were performed in accordance with National Institutes of Health Guide for the Care and Use of Laboratory Animals. One-month-old female swine (*Sus scrofa domesticus*, Yorkshire breed [8–10 kg]) were sedated with a mixture of intramuscular ketamine (20 mg/kg) and xylazine (2 mg/kg), followed by a mixture of inhaled isoflurane (~ 2% induction) and oxygen via nose cone to facilitate endotracheal intubation. Anesthesia was maintained with 0.5–1% isoflurane to provide adequate anesthesia while minimizing potential confounding toxicity and CBF changes associated with higher doses of isoflurane [22, 23].

Ventilator settings were as follows: tidal volume 10 mL/kg, positive end-expiratory pressure 6 cm H_2_O, and respiratory rate titrated to achieve end-tidal CO_2_ 38–42 mmHg to minimize potential confounding changes in CBF and acid-base status. Oxygen was titrated to maintain peripheral saturation of the blood greater than 92%. Arterial blood gas samples were used to evaluate baseline conditions of gas exchange, as well as at different stages during the experimental protocol. Pressure catheters were placed in the right femoral artery and vein for continuous measurements of right atrial and aortic pressures, allowing continuous calculation of coronary perfusion pressure. Specifically, high-fidelity micromanometer-tipped 4–5-Fr catheters were advanced from the femoral access sites to the right atrium and thoracic aorta—position was confirmed by fluoroscopy. Coronary perfusion pressure was measured by subtracting the right atrial pressure from the aortic pressure during diastole between chest compressions. Two unipolar pacing needles were briefly inserted transcutaneously into the right ventricle to initiate ventricular fibrillation for induction of cardiac arrest. Standard CPR with chest compressions commenced immediately after confirmation of induction of cardiac arrest by ventricular fibrillation. Prior to and during the experimental protocol, electrocardiogram, aortic blood pressure, right atrial pressure, pulse oximetry, and end tidal CO_2_ were displayed and recorded. A CPR quality-recording defibrillator (Zoll R Series Plus; Zoll Medical Corporation, Chelmsford, MA) was utilized during CPR and recorded chest compression rate (min^−1^) and depth (cm).

### Invasive cerebral hemodynamics

Under sterile conditions, two right-sided, 10-mm paramedian, small burr holes (2 mm and 3 mm) were made. The smaller burr hole was located 10 mm caudal to the coronal suture, while the larger was 10 mm cranial to the coronal suture. A bolt was placed into the largest burr hole, through which an intracerebral oxygenation and temperature probe (Licox, Integra Life Sciences, Plainsboro, NJ, USA) was inserted into the subcortical white matter and was equilibrated for a minimum of 30 min. A laser Doppler probe (Periflux 3000, Perimed, Ardmore, PA, USA) was secured in the smaller burr hole atop intact cortical dura for continuous measurements of CBF.

### Noninvasive cerebral hemodynamic measurements

Noninvasive quantification of cerebral tissue oxygen saturation (StO_2_, %) and CBF are achieved by leveraging the phenomenon of diffusive propagation of near-infrared light in tissue. The noninvasive technique of diffuse optical spectroscopy (DOS)—synonymous with near-infrared spectroscopy (NIRS), but used here to differentiate quantitative techniques—measures tissue absorption and scattering properties at multiple wavelengths which, given the known absorption properties of oxy- and deoxy-hemoglobin [24], are subsequently used to quantify the concentration of these predominating chromophores. Frequency-domain DOS (FD-DOS, i.e., FD-NIRS) advances upon existing clinical continuous-wave NIRS (CW-NIRS) techniques with the use of radio-frequency intensity-modulated laser sources [25]. This permits absolute quantification of the absorption and scattering properties of tissue from the change in light intensity and phase with respect to change in source-detector separation. Unlike CW-NIRS techniques such as those used in commercially available NIRS-based oximeter devices, FD-DOS does not rely on assumptions of baseline physiologic values to estimate tissue scattering properties [26]; rather, tissue scattering is directly measured. Thus oxy- and deoxy-hemoglobin concentration ([HbO_2_] and [Hb], respectively), total hemoglobin concentration (THC), and brain StO_2_ may be precisely quantified [27, 28].

CBF may also be quantified by the noninvasive diffuse optical technique of diffuse correlation spectroscopy (DCS) [29]. DCS examines the temporal fluctuations in detected intensity that are caused by the dynamic movement of light scatterers. As the predominant source of intensity fluctuations comes from the movement of red blood cells, the decay rate of the measured intensity autocorrelation function may be used to derive a cerebral blood flow index (BFI). DCS measurement of BFI has been shown to correlate with absolute CBF in children [30, 31]. In this animal model, we have previously demonstrated significant correlation of relative BFI with invasive laser Doppler measures of relative CBF during asphyxia and CPR [32]. Importantly, over the last decade, DCS has been increasingly used to quantify pathologic alterations in CBF in pediatric populations at high risk for neurological injury [29, 33–36].

Combining these two technologies and their respective output allows for continuous, noninvasive measurement of cerebral StO_2_, CBF, and critically, cerebral metabolic rate of oxygen [29, 37, 38]. In this study, animals were continuously monitored by noninvasive FD-DOS and DCS via an optical probe secured over the left frontal cortex. FD-DOS (110 MHz, Imagent, ISS, Inc.) quantification of tissue absorption and scattering properties were conducted at four wavelengths (690, 725, 785, and 830 nm) using multi-distance intensity and phase fitting versus source-detector separations of 1.5, 2.0, 2.5, and 3.0 cm. Measurements that did not achieve a Pearson correlation coefficient of 0.95 were omitted. Optical properties were then used to compute [HbO_2_], [Hb], THC, and StO_2_ assuming a cerebral water volume fraction of 75% [39, 40]. FD-DOS measurements were acquired at 10 Hz for 1 s interleaved with a single DCS frame. DCS calculation of BFI and relative CBF were derived from the average intensity autocorrelation function acquired over an integration time of 500 ms from a source-detector separation of 2.5 cm. The details of the DCS hardware and acquisition system have been previously detailed [32]. Importantly, the FD-DOS measurement of optical absorption and scattering properties, acquired immediately prior to each DCS frame, were used to derive BFI [41].

### Experimental protocol

Following calibration and synchronization of all monitoring equipment, animals were asphyxiated for 7 min, after which point they underwent ventricular fibrillation via two transcutaneous pacing needles (Supplementary Fig. S1). After confirmation of ventricular fibrillation on electrocardiogram, depth-guided CPR using electrode accelerometers was initiated to maintain a chest compression depth of 1/3 the antero-posterior chest depth at a rate of 100 compressions per minute while ventilating at 10 breaths per minute with 100% fraction of inspired oxygen. Brief 4-second interruptions in CPR every 2 min mimicked in-hospital pulse checks/rhythm analysis. An intravenous bolus of epinephrine (20 mcg/kg) was given via left femoral central venous catheter starting at 2 min into CPR (the mean time of epinephrine administration for pediatric in-hospital cardiac arrest) [42]. Subsequent doses of epinephrine were given every 4 min thereafter, consistent with Pediatric Advanced Life Support Guidelines. To ensure rigorous adherence to CPR targets, the Zoll R-series defibrillator with Real CPR Help® (Chelmsford, MA) and a metronome were used. After 10 min of CPR, defibrillation (5 J/kg) was attempted via external pads placed along the sternum and at the cardiac apex. The first defibrillation attempt was delayed to 10 min in order to allow a 10-min minimum period of time in which to perform CPR, and thereby test the hypotheses of the investigation. This duration of CPR is consistent with the median duration of CPR observed in actual pediatric in-hospital cardiac arrests [43]. CPR was continued until sustained ROSC of ≥ 20 min was achieved, or after an additional 10 min of resuscitation post-initial defibrillation attempt, after which time resuscitative efforts were discontinued (20-min maximum of CPR). Following ROSC, animals received protocolized intensive care unit treatment that has been previously described [44, 45] for a period of 4 h, after which time animals were humanely euthanized with potassium chloride (2 mEq/kg).

### Post-resuscitative protocolized care

To limit variability between groups and to simulate current clinical intensive care unit management, animals that achieved ROSC received protocolized intensive care unit treatment including (1) titration of analgesia, (2) titration of fraction of inspired oxygen to maintain systemic oxygen saturation 92–96% by pulse oximetry on the limb, (3) titration of ventilation to maintain end tidal CO_2_ 38–42 mmHg, and (4) initiation and titration of intravenous vasopressor infusions (epinephrine up to 1 mcg/kg/min) or isotonic fluids (0.9% normal saline up to 60 mL/kg) as necessary to maintain mean arterial pressure greater than 45 mmHg.

### Statistics

Continuous output from all systemic and cerebrovascular hemodynamic measurements, as well as FD-DOS/DCS measurements, was collected during asphyxia, CPR, and the post-ROSC period. All statistical analyses were performed in MATLAB, Release R2018a (Mathworks, Inc., Natick, MA, USA).

The primary questions posed in our analysis were (1) does each epinephrine dose have a significant acute effect on CBF and cerebral tissue oxygenation and (2) does the effect of each subsequent dose of epinephrine differ significantly from that of the first. To address the first question, the acute effect was defined as the difference between the post-dose mean, in the minute immediately following dose administration, and the pre-dose mean, in the minute immediately preceding dose administration. Data during chest compression pauses were omitted in the calculation of these mean values. Determination of significant dose effect was made by a Wilcoxon signed-rank test comparing the pre-dose mean to post-dose mean. To address the second question, the Wilcoxon signed-rank test was also used to compare each subsequent dose’s effect size to that of the first dose. Post hoc false discovery rate correction was performed to account for repeated comparisons (false discovery rate = 0.05) [46]. To quantify the peak hemodynamic response, a supplementary analysis was performed comparing the maximum post-dose value to the median pre-dose value within the minute immediately preceding dose administration. These summary results are reported as median followed by interquartile range in brackets ([IQR]).

To account for inter-animal variability of cerebrovascular hemodynamic measurement baselines, invasive and noninvasive variables were indexed relative to each animal’s pre-asphyxia baseline. The following invasive measurement variables were analyzed: systolic blood pressure, diastolic blood pressure, coronary perfusion pressure, relative brain tissue oxygen tension (rPbtO_2_), and relative CBF (rCBF, laser Doppler). The following noninvasive variables were analyzed: relative diffuse correlation spectroscopy (rDCS, i.e., BFI/noninvasive CBF), relative brain StO_2_ (rStO_2_), relative total hemoglobin concentration (rTHC), relative oxy-hemoglobin concentration (r [HbO2]), and relative deoxy-hemoglobin concentration (r [Hb]).

To assess correlation of noninvasive and invasive metrics of rCBF, the following methods were used. Asphyxia and CPR time period data were separately modeled due to the observation of discrepant, period-specific relationships between measurement modalities. For each subject, data were binned in 10% intervals with respect to invasive rCBF measurements. Thus, for the set of invasive rCBF points that fell within each 10% interval (e.g., 0–10%, 10–20%, etc.), the value of noninvasive rCBF was determined to be the mean of the corresponding noninvasive rCBF measures of those points. Correlation and linear relationships between binned invasive and noninvasive rCBF measurements were assessed using a linear mixed effects model with the non-invasive rCBF as the dependent variable and the invasive rCBF as the independent variable. This model incorporated subject-specific random intercept and slope to account for the within-subject clustering due to multiple measures taken on the same subject. The linear mixed-effects model was fitted using MATLAB function *fitlme*. The fitted model produces an adjusted *R*^2^ value that is equivalent to the *R*^2^ (i.e., coefficient of determination) in a regular linear regression model, which quantifies the correlation between the dependent and independent variables in a simple linear regression. Precision and limits of agreement between modalities were assessed by repeated measures Bland-Altman analysis. The Bland-Altman analysis was constructed in MATLAB. The calculation of repeated measures bias and limits of agreement were conducted as described by Bland et al. [47]

## Results

Overall, ROSC was achieved in 16/20 (80%) animals. Mean baseline conditions and physiologic changes during the experimental protocol as reflected by available arterial blood gases are provided in Supplementary Table S1. Eleven animals received 2 doses of epinephrine and achieved ROSC on the first defibrillation attempt (10 min of CPR). For the additional five animals who achieved ROSC, additional protocolized CPR, defibrillation, and epinephrine doses were required. Following ROSC, animals were not given additional doses of epinephrine. Two animals achieved ROSC following the third dose of epinephrine and subsequent defibrillation (12 min of CPR), and an additional 3 animals achieved ROSC following the fourth dose of epinephrine and subsequent defibrillation (14–18 min of CPR). No animal who received a fifth dose of epinephrine achieved ROSC.

Table 1 illustrates the effect of each epinephrine dose on all measures of cerebrovascular and systemic hemodynamics and oxygenation. Parameters with significance (*p* < 0.05) following false discovery rate adjustment are denoted with an asterisk. With the first epinephrine dose during CPR, rCBF and cerebral tissue oxygenation significantly increased by ~ 10%, as measured by either invasive or noninvasive measures (*p* < 0.01). By the third dose of epinephrine, there were no demonstrable increases in rCBF or cerebral tissue oxygenation. Invasive rPbtO_2_ increased 6.4% [0.6, 18.7] with the first epinephrine doses but did not increase with subsequent doses; rStO_2_ increased 7.9% [4.1, 12.6] and 3.4% [0.4, 7.0] with the first 2 epinephrine doses, respectively, but did not exhibit a significant effect with doses 3–5. rTHC, a surrogate measure of cerebral blood volume, also significantly increased only with the first 2 doses of epinephrine. These effects sizes were qualitatively smaller with a 3.0% [1.9, 6.4] increase in rTHC with the first dose and 1.1% [0.3, 2.0] increase with second dose. Similar incidence of significant dose effects was observed in systemic hemodynamics with the exception of diastolic blood pressure. Increases in systolic blood pressure and coronary perfusion pressure remained significant for the first two doses; systolic blood pressure exhibited a ~ 10 mmHg larger effect size compared to coronary perfusion pressure for both doses. In contrast, significant diastolic blood pressure effects were observed through the fourth dose. To also assess the significance of peak dose response, a table of analogous results comparing epinephrine pre-dose median to the post-dose max is shown in Supplementary Table S2 (mirroring Table 1). Only two comparisons differed in their determinations of significance after false discovery rate correction of multiple comparisons.

**Table 1:** Effects of intra-arrest epinephrine on cerebrovascular and systemic hemodynamics by dose

	Dose 1 (***n*** = 20)		Dose 2 (***n*** = 20)		Dose 3 (***n*** = 9)		Dose 4 (***n*** = 7)		Dose 5 (***n*** = 4)	
	Effect size	***p***	Effect size	***p***	Effect size	***p***	Effect size	***p***	Effect size	***p***
**(Δ, % Baseline)**										
**Invasive rCBF**	+ 8.2 [1.1, 22.4]	0.004*	+ 4.3 [1.3, 8.8]	0.002*	+ 3.5 [1.1, 8.0]	0.047	+ 0.9 [0.4, 2.3]	0.156	− 0.0 [− 0.7, 0.4]	0.875
**Non-invasive rCBF**	+ 17.3 [4.6, 23.6]	0.001*	+ 5.6 [0.4, 16.1]	0.015	+ 6.7 [0.5, 12.2]	0.156	+ 3.8 [− 2.3, 8.1]	0.438	− 3.7 [− 9.1, 3.6]	0.750
**rPbtO**_**2**_	+ 6.4 [0.6, 18.7]	0.001*	+ 0.3 [− 2.7, 5.0]	0.494	− 0.2 [− 6.1, 1.0]	0.426	− 0.2 [− 2.6, 0.3]	0.578	+ 0.1 [− 1.1, 0.5]	0.875
**rStO**_**2**_	+ 7.9 [4.1, 12.6]	< 0.001*	+ 3.4 [0.4, 7.0]	< 0.001*	+ 1.8 [− 0.8, 3.3]	0.129	− 1.1 [− 1.8, 2.0]	0.938	− 0.6 [− 2.3, 1.1]	0.625
**rTHC**	+ 3.0 [1.9, 6.4]	0.001*	+ 1.1 [0.3, 2.0]	0.007*	− 0.4 [− 2.3, 2.0]	> 0.999	+ 0.6 [− 0.5, 1.0]	0.578	− 0.7 [− 1.5, − 0.4]	0.125
**r [HbO**_**2**_**]**	+ 11.0 [5.9, 16.1]	< 0.001*	+ 6.0 [0.8, 9.6]	< 0.001*	+ 1.7 [− 1.8, 4.4]	0.250	− 0.9 [− 1.6, 3.3]	0.938	− 0.9 [− 3.1, 1.0]	0.625
**r [Hb]**	− 2.9 [− 12.1, 0.3]	0.006*	− 2.7 [− 6.2, 0.4]	0.012	− 1.4 [− 4.1, 0.2]	0.098	+ 0.5 [− 1.2, 1.4]	0.938	− 0.8 [− 1.9, − 0.2]	0.250
**(Δ, mmHg)**										
**SBP**	+ 17.6 [8.4, 52.0]	< 0.001*	+ 16.5 [6.4, 26.0]	< 0.001*	+ 21.0 [1.8, 34.7]	0.027	+ 9.7 [− 3.5, 18.1]	0.156	− 1.6 [− 3.2, 4.3]	0.875
**DBP**	+ 9.4 [5.0, 13.5]	< 0.001*	+ 5.5 [3.7, 9.0]	< 0.001*	+ 2.8 [1.1, 6.0]	0.012*	+ 2.4 [1.2, 5.4]	0.016*	+ 1.3 [− 0.7, 2.9]	0.625
**CPP**	+ 8.2 [3.9, 11.7]	< 0.001*	+ 4.5 [2.7, 8.2]	< 0.001*	+ 3.8 [0.9, 6.1]	0.020	+ 2.4 [1.2, 5.2]	0.016	+ 1.3 [− 0.7, 2.7]	0.625

Effect size reported as median [interquartile range]

*Abbreviations: Δ* change in value, *n* sample size, *rCBF* relative cerebral blood flow, *rPbtO*_*2*_ relative partial pressure of oxygen in brain tissue, *rStO*_*2*_ relative cerebral tissue oxygen saturation, *rTHC* relative total hemoglobin concentration, *r [HbO*_*2*_*]* relative concentration of oxy-hemoglobin, *r [Hb]* relative concentration of deoxy-hemoglobin, *SBP* systolic aortic blood pressure, *DBP* diastolic aortic blood pressure, *CPP* coronary perfusion pressure

*Adjusted *p* < 0.05 after false discovery rate correction for repeated comparisons

**Fig. 1:**
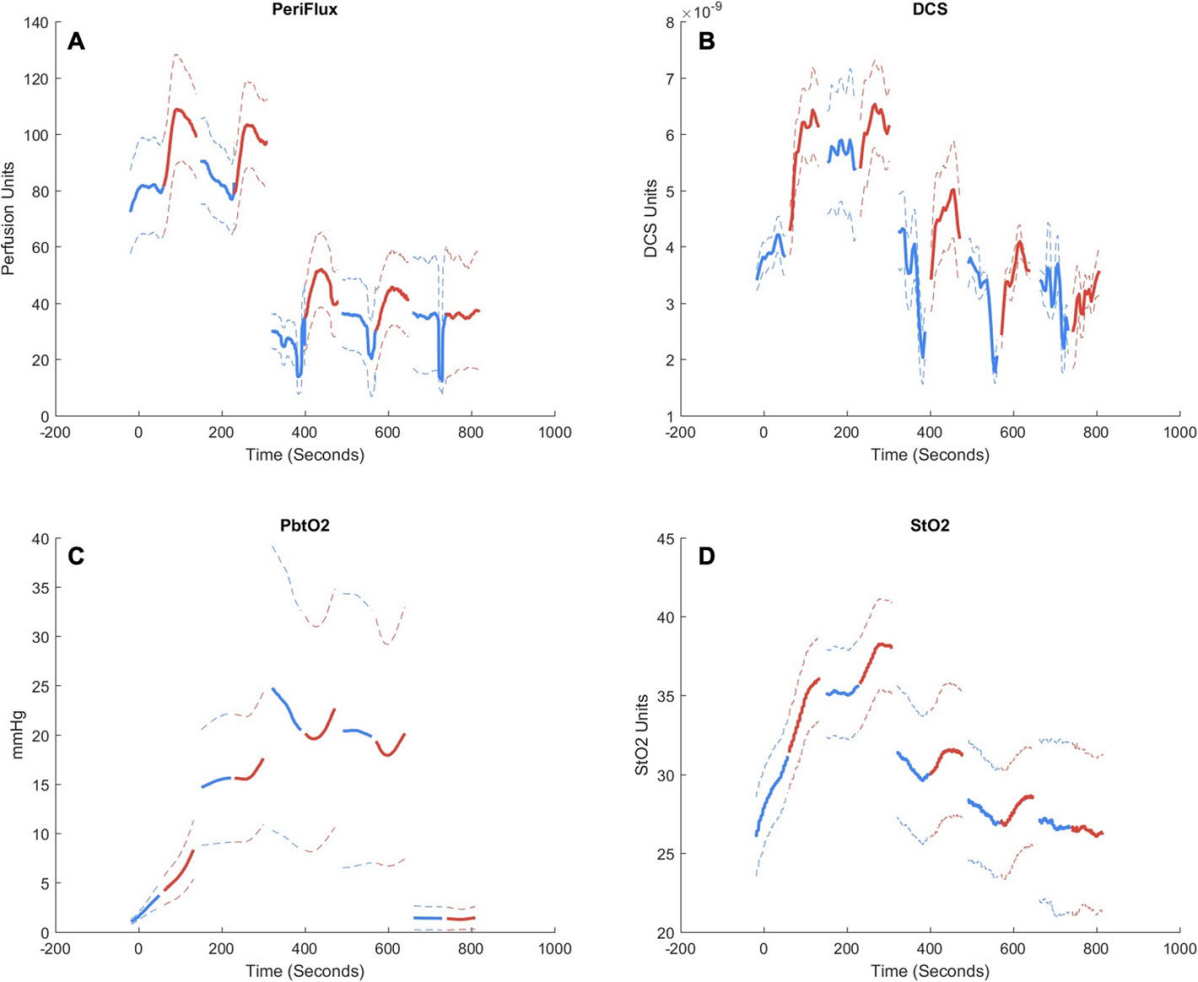
Time-series plots of aggregate subject data comparing effects of each epinephrine dose on cerebrovascular hemodynamics. Blue indicates pre-epinephrine values, and red indicates post-epinephrine values. **a** Invasive measurements of relative cerebral blood flow (CBF, Periflux laser Doppler). **b** Noninvasive measurements of relative CBF (diffuse correlation spectroscopy, DCS). **c** Invasive measurements of relative cerebral tissue oxygenation (PbtO_2_). **d** Noninvasive measurements of relative cerebral tissue oxygenation (StO_2_). Note, data during pauses were omitted from analysis

**Fig. 2:**
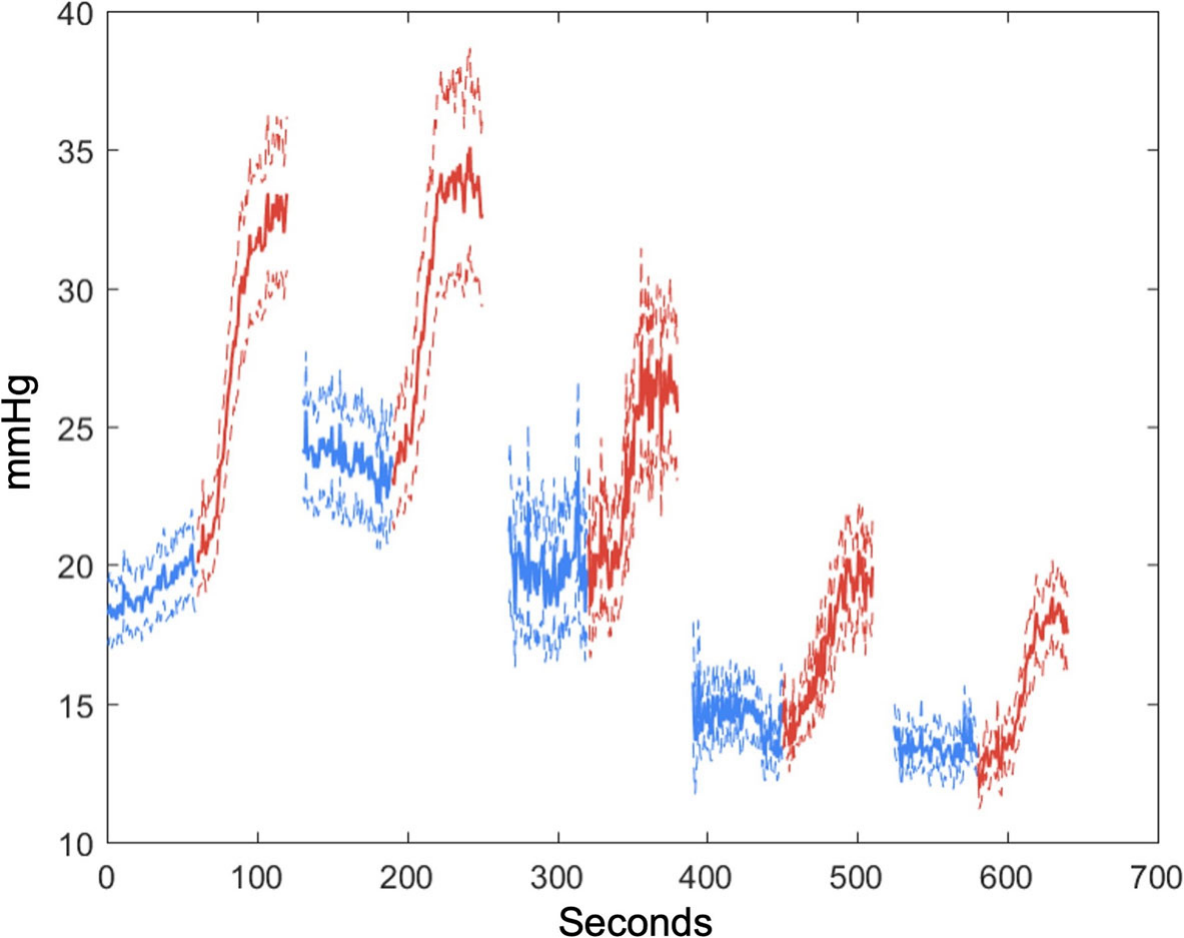
Diastolic blood pressure (DBP) effects of epinephrine per dose. While there is a significant effect of epinephrine in each dose, this effect is only sufficient to generate a DBP of greater than 30 mmHg in the first 2 doses. This threshold value was selected because of the previously demonstrated survival benefit associated with generating and maintaining a DBP > 30 mmHg during CPR in children [48]

Subsequent change in dose effect size, compared versus the first epinephrine dose effect size, is demonstrated in Table 2. There was diminished efficacy in the second dose for rCBF, rTHC, and blood pressure. In animals who required prolonged resuscitation (and therefore received greater than 3 doses of epinephrine), we observed no significant change in dose efficacy in these variables for subsequently repeated doses. Dose efficacy for cerebral oxygenation parameters (rStO2, rPbtO_2_, r [HbO_2_]) was significantly diminished by the second dose, with persistently diminished efficacy in rStO_2_ and rHbO_2_ in animals who required prolonged resuscitation (received greater than 3 doses). There was no significant change in dose effect for the fifth dose, but statistical power was limited. Following false discovery rate adjustment, we observed no significant changes in dose efficacy for rCBF, rTHC, and blood pressure. A table of analogous results comparing peak epinephrine dose effect sizes calculated using pre-dose median and post-dose max are shown in Supplementary Table S3. Raw time series data demonstrating the effects of epinephrine doses on noninvasive and invasive measurements of both rCBF and cerebral tissue oxygenation are depicted in Fig. 1, and the time series for diastolic blood pressure are depicted in Fig. 2 [48].

**Table 2:** Change in dose efficacy with repeated doses of intra-arrest epinephrine, comparison to dose 1

	Dose 2 (***n*** = 20)		Dose 3 (***n*** = 9)		Dose 4 (***n*** = 7)		Dose 5 (***n*** = 4)	
	Δ Effect size	***p***	Δ Effect size	***p***	Δ Effect size	***p***	Δ Effect size	***p***
**(Δ, % Baseline)**								
**Invasive rCBF**	− 3.8 [− 12.0, 0.6]	0.058	− 4.2 [− 4.8, 0.7]	0.469	− 2.7 [− 8.2, − 0.7]	0.219	− 2.2 [− 6.1, 2.6]	0.625
**Non-invasive rCBF**	− 3.0 [− 13.7, 1.0]	0.098	− 5.0 [− 11.0, 4.9]	0.375	− 1.3 [− 4.1, 0.3]	0.438	− 10.6 [− 14.1, 6.1]	0.750
**rPbtO**_**2**_	− 5.4 [− 22.0, 0.6]	0.044	− 1.6 [− 26.0, 1.9]	0.426	−1.6 [− 6.1, 1.5]	0.578	−2.7 [− 4.7, − 0.5]	0.250
**rStO**_**2**_	− 3.6 [− 10.4, 0.7]	0.005*	− 6.0 [− 8.9, − 2.9]	0.008*	− 5.3 [− 6.6, − 4.2]	0.016*	− 5.0 [− 7.5, − 3.9]	0.125
**rTHC**	− 1.9 [− 5.7, 1.3]	0.030	− 2.9 [− 5.6, 1.6]	0.250	− 1.5 [− 4.6, 1.5]	0.578	− 1.7 [− 4.8, 0.2]	0.375
**r [HbO**_**2**_**]**	− 3.9 [− 12.1, 0.1]	0.005*	− 7.6 [− 13.3, − 2.2]	0.004*	− 7.3 [− 8.0, − 4.5]	0.016*	− 4.8 [− 9.1, − 4.3]	0.125
**r [Hb]**	− 0.4 [− 3.1, 7.4]	0.351	+ 1.1 [− 0.7, 10.8]	0.301	+ 2.5 [0.0, 7.7]	0.109	+ 1.2 [− 1.3, 5.3]	0.625
**(Δ, mmHg)**								
**SBP**	− 5.9 [− 24.2, 3.0]	0.062	+ 1.8 [− 16.4, 12.1]	> 0.999	− 2.1 [− 10.6, 7.4]	0.578	− 10.7 [− 36.9, 1.5]	0.375
**DBP**	− 0.6 [− 5.8, 1.0]	0.117	− 7.3 [− 8.1, 1.0]	0.203	− 2.5 [− 5.5, 1.2]	0.297	− 3.2 [− 10.5, 1.3]	0.625
**CPP**	− 1.0 [− 5.7, 1.2]	0.086	− 4.4 [− 7.4, 1.8]	0.359	− 1.5 [− 4.7, 1.6]	0.469	− 2.7 [− 8.9, 1.3]	0.375

Effect Size reported as median [interquartile range]

*Abbreviations: Δ* change in value, *n* sample size, *rCBF* relative cerebral blood flow, *rPbtO*_*2*_ relative partial pressure of oxygen in brain tissue, *rStO*_*2*_ relative cerebral tissue oxygen saturation, *rTHC* relative total hemoglobin concentration, *r [HbO*_*2*_*]* relative concentration of oxy-hemoglobin, *r [Hb]* relative concentration of deoxy-hemoglobin, *SBP* systolic aortic blood pressure, *DBP* diastolic aortic blood pressure, *CPP* coronary perfusion pressure

*Adjusted *p* < 0.05 after false discovery rate correction for repeated comparisons

**Fig. 3:**
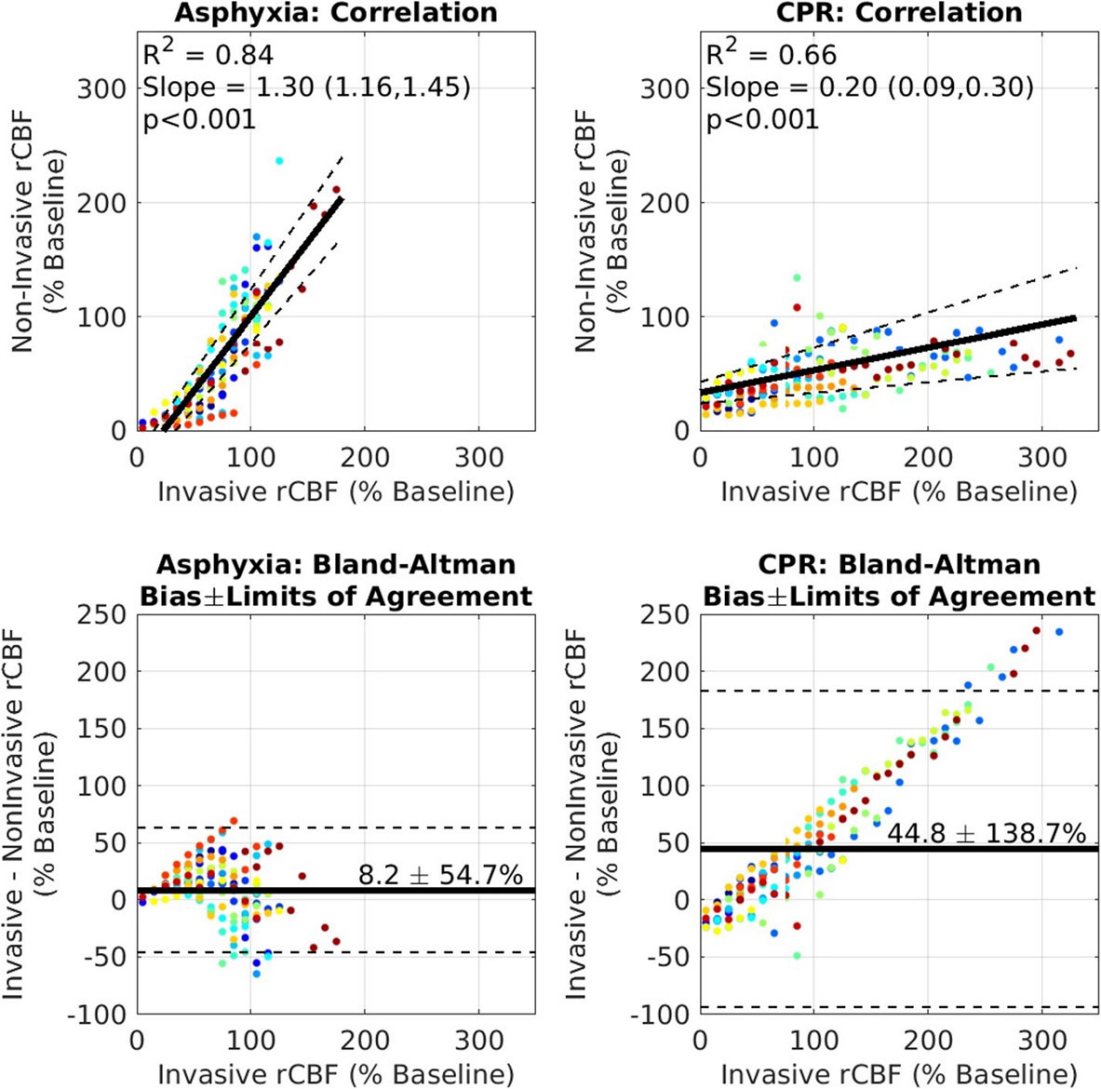
Linear correlation and Bland-Altman agreement plots. Plots for noninvasive (DCS) versus invasive (laser Doppler) measurements of relative cerebral blood flow (rCBF). During periods of both asphyxia and cardiac arrest, comparisons with noninvasive rCBF and invasive rCBF were validated. Each study subject is represented by a unique color

Figure 3 depicts validation of noninvasive rCBF by comparison with invasive rCBF during both the asphyxia period and CPR period of the experiments. Noninvasive and invasive measures of rCBF were strongly correlated (*R*^2^ = 0.84) during asphyxia with a highly significant slope effect of 1.3 (95% CI = 1.16–1.45, *p* < 0.001). During the asphyxia period, invasive measurements demonstrated a mean bias and 95% limits of agreement of 8.2% ± 54.7% in comparison with noninvasive measurements. The significant slope effect was also observed during CPR (0.20, 95% CI = 0.09–0.30, *p* < 0.001), with *R*^2^ = 0.66. Invasive rCBF measurements tended to be higher than noninvasive measurements at values greater than 50% of baseline rCBF. This skew was also evident in Bland-Altman analysis of agreement, where invasive measurements demonstrated a mean bias and 95% limits of agreement of 44.8% ± 138.7% in comparison with noninvasive measurements.

## Discussion

These data establish that CBF and cerebral tissue oxygenation increased by > 10% with the first epinephrine dose during CPR, as measured by each of the invasive and noninvasive measures (*p* < 0.001). Epinephrine’s effect on CBF and cerebral tissue oxygenation decreased with subsequent doses, and by the fifth dose of epinephrine, there were no demonstrable increases in CBF or cerebral tissue oxygenation. In addition, the noninvasive measurements of CBF and cerebral tissue oxygenation directly correlated with invasive measurements, providing support for further translational and clinical investigations to determine the value of real-time noninvasive CBF and/or cerebral tissue oxygenation DCS/DOS monitoring during CPR.

Much of the debate about neurologic effects of epinephrine administration during CPR emanates from previous translational laboratory studies with varied methodology. Many robust laboratory studies have established and confirmed that epinephrine improves overall CBF using microsphere quantification of blood flow in multiple regions of the brain [13–15, 49–51]. In contrast, Ristagno and colleagues demonstrated decreases in cerebral microvascular flow using orthogonal polarization spectral imaging-based measurements [18, 19]. While orthogonal polarization spectral imaging provides quantitative and qualitative assessment of capillary blood flow within its focal range, the range is limited to an area 1 mm below the cortical surface. Other investigators have shown variable effects of epinephrine during CPR on carotid blood flow in large animal models [19, 20]. However, these findings are confounded by the well-established large contribution of carotid blood flow to the external carotid artery in canines and swine, such that carotid flow does not necessarily correlate with CBF, as well as the decreased external carotid blood flow due to systemic circulation vasoconstriction from epinephrine during CPR [52].

Our data establish that the first epinephrine dose during CPR increases median CBF by 11.5% [interquartile range (IQR) 3.4–29.3%] with invasive laser Doppler technique and by 20.4% [IQR 12.9–44.5%] with noninvasive DCS technique. These results are consistent with the findings of Johannson et al. that demonstrated significantly increased CBF with both continuous and bolus doses of epinephrine in a swine ventricular fibrillation cardiac arrest model using laser Doppler quantification of cortical CBF [16]. The increased blood flow in our study was associated with increased cerebral tissue oxygenation by 10.0% [IQR 1.5–34.1%] with invasive PbtO_2_ technique (Licox) and by 11.0% [IQR 5.8–18.7%] by noninvasive DOS StO_2_. These cerebral tissue oxygenation data are consistent with recent swine CPR observations from Nosrati and colleagues demonstrating that epinephrine boluses increased noninvasive StO_2_ by hyperspectral NIRS (QE65000 Ocean Optics, Dunedin, FL) [17].

We demonstrated that the effect of epinephrine on CBF and cerebral tissue oxygenation decreased with subsequent doses. Whereas the first 2 doses of epinephrine significantly improved CBF and cerebral tissue oxygenation, the effects waned following the second dose, and no effects were demonstrable by the fifth dose. Notably, previous studies have similarly shown progressive decreases in the effect of epinephrine on cerebral perfusion pressures, CBF, and/or cerebral tissue oxygenation with repeated doses [17, 21, 49, 50, 53, 54].

This experiment also provided a model for validating noninvasive measurements of CBF and tissue oxygenation with invasive measurements during periods of asphyxia and CPR. During asphyxia, noninvasive CBF appeared to be more sensitive to hemodynamic changes compared to invasive measurements. This may be a result of superficial tissue contamination, whereby, superficial tissue exhibits a more rapid hyperemic and subsequent ischemic response than underlying cerebral tissue. At lower CBF values, greater agreement between the two modalities was observed. During CPR, both modalities may have been influenced by motion artifacts. For both modalities, the measured signal is based on decorrelation of the intensity of back-scattered light. At steady state, the light is predominantly scattered by moving red blood cells. Thus, the more rapidly red blood cells move, the faster this decorrelation occurs. However, if the surrounding tissue is also in motion, additional decorrelation occurs that will be erroneously attributed to red blood cell motion. Given the smaller pathlength of light used by the laser Doppler modality (a single-scatter measurement) versus noninvasive DCS (a multiple-scatter measurement), DCS may be less affected by bulk tissue movement. Despite the inherent challenge of motion artifact and differential tissue saturation during asphyxia and CPR, our data still provide strong evidence of correlation between noninvasive and invasive measurements of CBF and cerebral tissue oxygenation that holds considerable promise for the use of noninvasive measurements to guide future therapy during CPR. Thus, our study provides novel, multimodal, invasive, and noninvasive measurements of epinephrine’s effects on cerebrovascular hemodynamics and cerebral tissue oxygenation during CPR. These novel data reveal good correlation of noninvasive and invasive measurements of CBF and cerebral tissue oxygenation during CPR in an asphyxia cardiac arrest and CPR model.

Because there are several examples of apparently conflicting data in the literature that employed different methods to assess cerebrovascular hemodynamics in response to epinephrine, it is important to consider the inherent limitations and advantages associated with different imaging and measurement modalities. In a study by Hardig et al., CBF was not directly measured when concluding that epinephrine boluses decreased cerebral perfusion. Instead, CBF was inferred from changes in cerebral oximetry values using a commercially available CW-NIRS-based oximeter [21]. Although concomitant decreases in cerebral oximetry values and decreases in CBF have been previously reported, there are several limitations to the CW-NIRS technology that makes interpreting these data challenging [55, 56]. While a detailed discussion on the differences between FD-DOS (i.e., FD-NIRS) and CW-NIRS is beyond the scope of this manuscript, certain points should be considered. Firstly, the limitations of CW-NIRS in providing accurate data on cerebral oximetry during physiologic extremes are well documented [57]. These limitations are particularly relevant during the severe hypoxic-ischemic conditions associated with cardiac arrest [58, 59]. Secondly, compared to CW-NIRS, FD-NIRS-based measurements provide absolute quantification of individual hemoglobin species (oxy- and deoxy-hemoglobin) which permits more accurate, absolute measurement of cerebral oximetry (StO_2_) [60]. Moreover, FD-NIRS has been clinically validated against gold standard measurements of tissue oxygen saturation and provide absolute values [61, 62]. Meanwhile, although DCS is a relatively new measurement modality for CBF, it has been rigorously validated against numerous clinical gold standard measures of CBF, including fluorescent microspheres, magnetic resonance imaging, and Xenon-enhanced computerized tomography in a variety of human and animal models [29, 32]. Our noninvasive optical data are congruent with more widely accepted, clinically used (albeit in contexts outside of CPR) invasive measurements of CBF (laser Doppler) and cerebral tissue oxygenation (PbtO_2_). For these reasons, we believe that our data offers a more complete assessment of epinephrine’s effects on cerebrovascular hemodynamics than studies where only one modality was used and CBF was inferred rather than measured.

In contrast to previous studies, the imaging modalities used in our current experiment allow for an assessment of deeper cortical perfusion and oxygenation. The hybrid DCS/DOS probe that we used to quantify CBF and cerebral tissue oxygenation has a source-detector length that is optimized to take measurements at 8–12.5 mm. Both skull and scalp thickness were recorded in all animals during necropsy, and the resulting range was 5–6.5 mm. As such, our noninvasive measurements were measuring CBF at least 2 mm into the cerebral cortex, making them deeper than previous experiments [18, 19], which possibly represents a different network of microvasculature than may be encountered along the cortical surface. Our invasive measurements were made at a similar depth. The laser Doppler probe that was used has a sensitivity < 5 mm from the originating signal; since it was placed along the dura mater, this means that our CBF measurements were taken within the first 5 mm of the cerebral cortex. Our cerebral PbtO_2_ measurements were attained at 20 mm below the dura using the proprietary bolt to ensure consistency. These differences in modalities are important for interpreting our findings in the context of other seemingly conflicting studies [18, 19]. Because our measurements assess deeper cortical hemodynamics, it may be that epinephrine has differential effects on deeper cortical blood flow compared to the cortical surface, but further research is needed to delineate epinephrine’s effects on different brain regions during CPR. The imaging modalities used in this study are directed primarily at subcortical gray and white matter. Injury patterns in children following cardiac arrest vary according to the extent and mechanism of injury [63]. In addition to cortical injury, vulnerable deep structures include the hippocampus and reticular thalamus, which are metabolically active and are watershed areas of cerebral perfusion [64–68]. While the exact pattern of neuronal injury distribution in pediatric cardiac arrest has not been elucidated, these studies suggest that the regions most vulnerable to injury in the anoxia, ischemia, and subsequent reperfusion associated with cardiac arrest lie in structures that are deeper than the superficial cortex.

As such, a more intricate understanding of CBF regulation and the cerebrovascular effects of epinephrine will likely be important to improve patient survival that maintains freedom from neurologic morbidity after cardiac arrest. This is highlighted in several clinical trials demonstrating that higher doses of epinephrine may improve the incidence of ROSC in cardiac arrest, while similar benefits are not observed in neurologic outcomes [3, 5, 69–73]. Although some animal studies have previously demonstrated decreasing effects with subsequent epinephrine doses in cardiac arrest models [21, 53, 54], our results indicate a more robust interval of improvement in CBF and cerebral oxygenation prior to plateaus, particularly in deeper cortical structures. We importantly, and more definitively, demonstrate that epinephrine does in fact improve CBF and cerebral oxygenation when administered shortly after cardiac arrest in a pediatric in-hospital cardiac arrest model.

It is noteworthy that the vast majority of extant animal studies on epinephrine and cerebral hemodynamics were performed in adult swine [18–21]. Because the development of cerebrovascular autoregulatory mechanisms is an ongoing field of research, there may be important differences between the pediatric and adult responses to epinephrine during cardiac arrest and CPR. Cardiac arrest, ischemia, and reperfusion generate many disruptive pathophysiologic changes that likely affect cerebrovascular autoregulation and the extent to which the cerebral vasculature can compensate may differ in these populations. Our study is more representative of a pediatric in-hospital cardiac arrest model with a relatively short period of ischemia prior to reperfusion, whereas other models are more characteristic of adult and/or out-of-hospital cardiac arrests with prolonged and variable durations of time prior to the initiation of CPR and pharmacologic resuscitation. Notably, the median time to the first dose of epinephrine for adult out-of-hospital cardiac arrest is 20 min [3], whereas in translational laboratory studies intended to model this clinical problem often provide epinephrine after much briefer periods of time [53, 54]. Therefore, it remains to be studied if delayed administration of epinephrine will similarly improve CBF and cerebral oxygenation in adult and/or out-of-hospital cardiac arrest models (as demonstrated in our investigation), and if such increases in CBF and cerebral oxygenation are translatable to improved neurologic outcomes in any population. Such important model differences should be considered when interpreting our findings and others’, as differences in study design may not necessarily be translatable to all models of cardiac arrest.

In light of these considerations, our study has several limitations. This investigation was an observational study in which the first dose of epinephrine was provided after 2 min of chest compressions, consistent with the published mean time to epinephrine administration for pediatric in-hospital cardiac arrests [42], rather than after 4 min as suggested by international guidelines. Similarly, although international guidelines recommend epinephrine doses of 10 mcg/kg in humans, this investigation used a dose of 20 mcg/kg, which is the standard swine dose for CPR studies over the last 40 years in many laboratories in the USA and Europe [74–78], although a few leading swine CPR investigators have used doses of 30–45 mcg/kg [18, 79, 80]. Because of the observational nature of this study, there was no placebo group. In addition, there was no assessment of differing doses of epinephrine, and no assessment of alternative vascular routes (i.e., peripheral IV, intraosseous, etc.). The clinical scenario represented by this translational study was that of pediatric, in-hospital cardiac arrest, the majority of which are asphyxial in nature. Thus, our findings may not be applicable to out-of-hospital arrests or primary ventricular fibrillation events. Because these animals were previously healthy prior to asphyxia, cardiac arrest, and CPR, our findings may not represent the myriad confounding metabolic and hemodynamic effects of comorbid conditions that many patients experience prior to a cardiac arrest event.

## Conclusions

This study marks an important advancement in our understanding of epinephrine’s cerebrovascular effects and supports further investigation of noninvasive measurements of key neurological health parameters during CPR. Despite widespread use, the exact mechanisms of epinephrine’s effects on CBF and cerebral oxygenation, which are crucial to a patient achieving ROSC free of neurologic injury, remain incompletely understood. These results suggest that epinephrine increases CBF and cerebral tissue oxygenation, but that effects wane with repeated doses. The inherent challenges associated with continuous neuromonitoring during CPR have hindered the development of optimal monitoring and treatments to ensure adequate brain perfusion and resuscitation during CPR. The methods outlined in this investigation may help to establish real-time neurological monitoring that can individualize, optimize, and improve resuscitation strategies across all patient populations.

## Supplementary information

**Additional file 1: Supplementary Figure S1.** Graphic Depiction of Experimental Protocol and Timeline of Events. The endotracheal tube (ETT) was clamped and piglets were asphyxiated for 7 min prior to ventricular fibrillation induction. After cardiac arrest, depth-guided cardiopulmonary resuscitation (CPR) was performed using electrode accelerometers to maintain a chest compression depth of 1/3 the antero-posterior chest depth at a rate of 100/min. Epinephrine was first administered after 2 min (the mean time to epinephrine administration during pediatric in-hospital cardiac arrest). The standard swine dose of epinephrine (20mcg/kg) was used. After the first dose, epinephrine was given every 4 min, consistent with international guidelines until there was sustained return of spontaneous circulation, or a maximum 20 min of CPR.

**Additional file 2.**

**Additional file 3.**

**Additional file 4.**

## Data Availability

The datasets used and/or analyzed during the current study are available from the corresponding author on reasonable request.

## Abbreviations

BFI: Blood flow index
CBF: Cerebral blood flow
CPR: Cardiopulmonary resuscitation
CW-NIRS: Continuous-wave near-infrared spectroscopy
DOS: Diffuse optical spectroscopy
FD: Frequency domain
[Hb]: Deoxy-hemoglobin concentration
[HbO_2_]: Oxy-hemoglobin concentration
NIRS: Near-infrared spectroscopy
PbtO2: Partial pressure of oxygen in brain tissue
ROSC: Return of spontaneous circulation
StO_2_: Tissue oxygen saturation
THC: Total hemoglobin concentration
