# Supplementary figures and images for: Epinephrine’s effects on cerebrovascular and systemic hemodynamics during cardiopulmonary resuscitation
Source: Crit Care. 2020 Sep 29;24:583. doi: 10.1186/s13054-020-03297-4 (PMC7522922; doi:10.1186/s13054-020-03297-4)

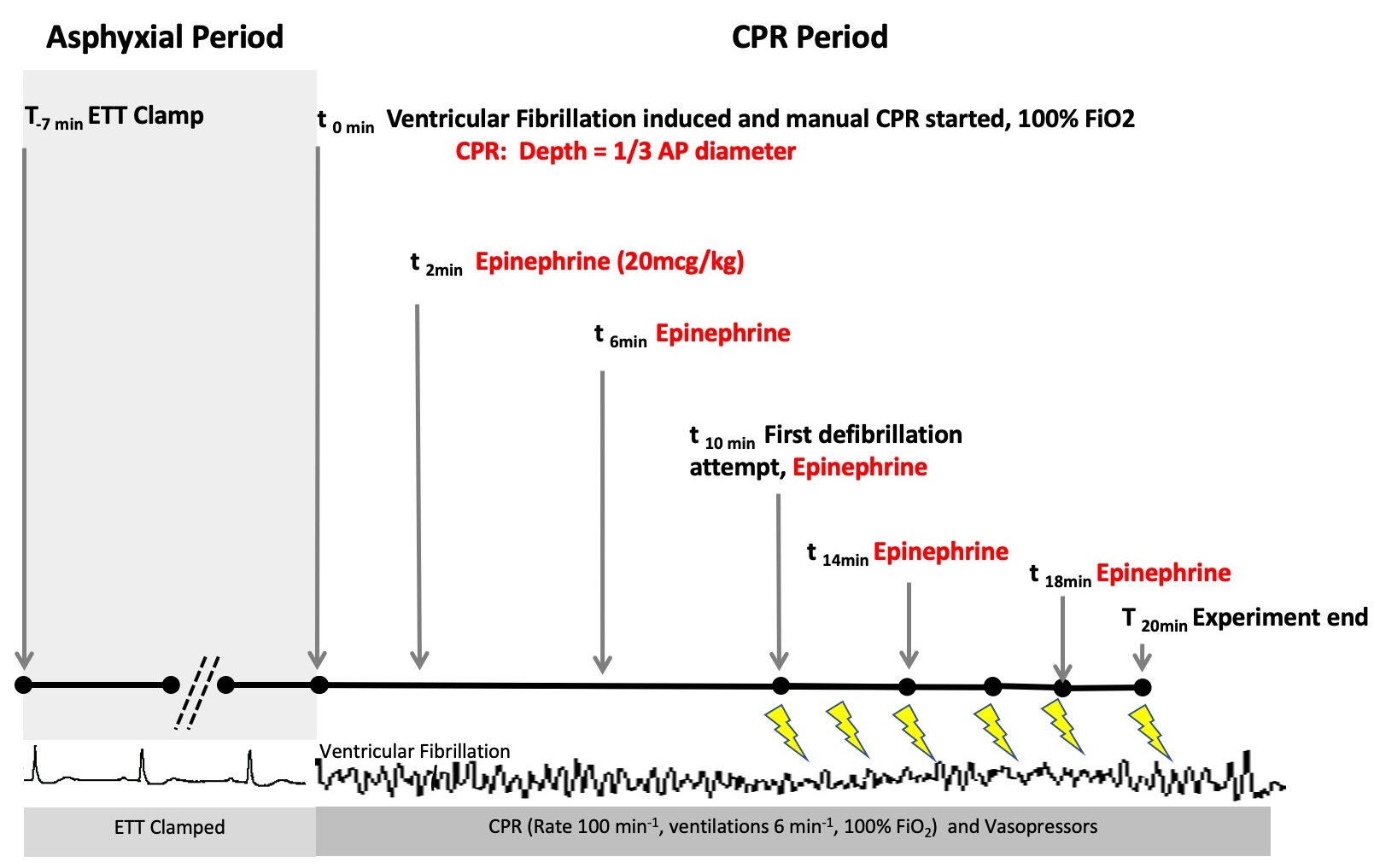

Supplement: Supplementary file 1 — Additional file 1: Supplementary Figure S1. Graphic Depiction of Experimental Protocol and Timeline of Events. The endotracheal tube (ETT) was clamped and piglets were asphyxiated for 7 min prior to ventricular fibrillation induction. After cardiac arrest, depth-guided cardiopulmonary resuscitation (CPR) was performed using electrode accelerometers to maintain a chest compression depth of 1/3 the antero-posterior chest depth at a rate of 100/min. Epinephrine was first administered after 2 min (the mean time to epinephrine administration during pediatric in-hospital cardiac arrest). The standard swine dose of epinephrine (20mcg/kg) was used. After the first dose, epinephrine was given every 4 min, consistent with international guidelines until there was sustained return of spontaneous circulation, or a maximum 20 min of CPR. [file 13054_2020_3297_MOESM1_ESM.jpg]
