# Supplementary material for: Epinephrine’s effects on cerebrovascular and systemic hemodynamics during cardiopulmonary resuscitation
Source: Crit Care. 2020 Sep 29;24:583. doi: 10.1186/s13054-020-03297-4 (PMC7522922; doi:10.1186/s13054-020-03297-4)
Supplement: Supplementary file 2 — Additional file 2. [file 13054_2020_3297_MOESM2_ESM.docx]

| **Table S1: Mean Physiologic Conditions of Study Subjects Reflected by Arterial Blood Gases** | | | | | | | | | | | | |
| --- | --- | --- | --- | --- | --- | --- | --- | --- | --- | --- | --- | --- |
|  | **Baseline pH** | **Baseline PCO_2_** | **Baseline O_2_** | **Asphyxia pH** | **Asphyxia PCO_2_** | **Asphyxia PO_2_** | **CPR pH** | **CPR PCO_2_** | **CPR PO_2_** | **Post pH** | **Post PCO_2_** | **Post PO_2_** |
| **Overall** | | | | | | | | | | | | |
| **Mean** | 7.44 | 40.46 | 112.35 | 7.23 | 61.27 | 39.50 | 7.29 | 45.27 | 209.73 | 7.28 | 43.41 | 119.64 |
| **St Dev** | 0.05 | 3.60 | 15.36 | 0.14 | 18.45 | 44.53 | 0.15 | 11.68 | 162.38 | 0.13 | 6.13 | 33.49 |
| **n** | 26 | 26 | 26 | 22 | 22 | 22 | 15 | 15 | 15 | 22 | 22 | 22 |
| **0 Epinephrine Dose** | | | | | | | | | | | | |
| **Mean** | 7.44 | 43.33 | 105.17 | 7.45 | 41.17 | 111.33 | 7.45 | 42.17 | 183.33 | 7.46 | 41.00 | 131.67 |
| **St Dev** | 0.05 | 4.07 | 13.70 | 0.04 | 1.86 | 6.34 | 0.03 | 3.13 | 153.31 | 0.04 | 2.08 | 23.55 |
| **n** | 0.05 | 6 | 6 | 6 | 6 | 6 | 6 | 6 | 6 | 6 | 6 | 6 |
| **2 Epinephrine Doses** | | | | | | | | | | | | |
| **Mean** | 7.41 | 40.40 | 112.30 | 7.14 | 64.63 | 14.50 | 7.16 | 47.17 | 256.50 | 7.23 | 42.67 | 117.22 |
| **St Dev** | 0.04 | 2.91 | 15.77 | 0.03 | 18.23 | 7.05 | 0.07 | 13.32 | 161.43 | 0.06 | 6.46 | 37.08 |
| **n** | 10 | 10 | 10 | 8 | 8 | 8 | 6 | 6 | 6 | 9 | 9 | 9 |
| **3 Epinephrine Doses** | | | | | | | | | | | | |
| **Mean** | 7.47 | 37.33 | 129.67 | 7.15 | 58.50 | 13.00 | 7.28 | 36.00 | 233.50 | 7.25 | 42.00 | 97.00 |
| **St Dev** | 0.01 | 0.47 | 9.46 | 0.00 | 10.50 | 8.00 | 0.02 | 2.00 | 159.50 | 0.05 | 1.41 | 7.12 |
| **n** | 3 | 3 | 3 | 2 | 2 | 2 | 2 | 2 | 2 | 3 | 3 | 3 |
| **4 Epinephrine Doses** | | | | | | | | | | | | |
| **Mean** | 7.51 | 38.00 | 113.67 | 7.18 | 81.00 | 16.00 | 7.13 | 71.00 | 40.00 | 7.17 | 47.00 | 108.00 |
| **St Dev** | 0.04 | 0.82 | 7.41 | 0.07 | 9.00 | 4.00 |  |  |  | 0.04 | 6.38 | 32.78 |
| **n** | 3 | 3 | 3 | 2 | 2 | 2 | 1 | 1 | 1 | 3 | 3 | 3 |
| **5 Epinephrine Doses** | | | | | | | | | | | | |
| **Mean** | 7.42 | 41.50 | 117.50 | 7.18 | 78.50 | 3.00 |  |  |  |  |  |  |
| **St Dev** | 0.04 | 3.50 | 13.50 | 0.02 | 1.50 | 2.00 |  |  |  |  |  |  |
| **n** | 2 | 2 | 2 | 2 | 2 | 2 | 0 | 0 | 0 | 0 | 0 | 0 |
| Mean arterial blood gas values of baseline samples taken prior to the experimental protocol, as well as during asphyxiation, high-quality cardiopulmonary resuscitation (CPR), and post-experimental. | | | | | | | | | | | | |
