# Supplementary material for: Epinephrine’s effects on cerebrovascular and systemic hemodynamics during cardiopulmonary resuscitation
Source: Crit Care. 2020 Sep 29;24:583. doi: 10.1186/s13054-020-03297-4 (PMC7522922; doi:10.1186/s13054-020-03297-4)
Supplement: Supplementary file 3 — Additional file 3. [file 13054_2020_3297_MOESM3_ESM.docx]

| **Table S2: Effects of Intra-Arrest Epinephrine on Cerebrovascular and Systemic Hemodynamics by Dose** | | | | | | | | | | |
| --- | --- | --- | --- | --- | --- | --- | --- | --- | --- | --- |
|  | **Dose 1 (n=20)** |  | **Dose 2 (n=20)** |  | **Dose 3 (n=9)** |  | **Dose 4 (n=7)** |  | **Dose 5 (n=4)** |  |
| **(Δ, % Baseline)** | **Effect Size** | **p** | **Effect Size** | **p** | **Effect Size** | **p** | **Effect Size** | **p** | **Effect Size** | **p** |
| **Invasive rCBF** | +11.5 [3.4, 29.3] | <0.001* | +7.2 [2.0, 13.9] | <0.001* | +7.4 [2.1, 14.2] | 0.016 | +1.9 [1.0, 4.4] | 0.031 | +0.8 [0.3, 3.0] | 0.250 |
| **Non-Invasive rCBF** | +20.4 [12.9, 44.5] | <0.001* | +12.0 [7.8, 25.1] | 0.005* | +15.1 [5.0, 18.5] | 0.016 | +8.2 [3.8, 12.6] | 0.062 | +1.4 [-4.5, 7.0] | 0.750 |
| **rPbtO_2_** | +10.0 [1.5, 34.1] | <0.001* | +2.5 [-1.7, 8.4] | 0.044 | -0.2 [-3.1, 2.0] | 0.734 | +0.0 [-1.8, 1.0] | 0.812 | +0.4 [-0.3, 1.5] | 0.625 |
| **rStO_2_** | +11.0 [5.8, 18.7] | <0.001* | +6.4 [2.5, 10.9] | <0.001* | +4.2 [0.7, 6.0] | 0.020 | +0.8 [-0.3, 4.5] | 0.297 | +0.6 [-1.0, 2.8] | 0.625 |
| **rTHC** | +4.8 [2.6, 9.1] | <0.001* | +1.6 [0.7, 3.1] | 0.001* | +1.1 [-1.6, 2.8] | 0.570 | +1.6 [-0.2, 2.6] | 0.156 | -0.4 [-0.8, 0.1] | 0.375 |
| **r[HbO_2_]** | +16.1 [8.7, 24.5] | <0.001* | +10.1 [3.0, 14.2] | <0.001* | +5.5 [0.5, 6.7] | 0.027 | +2.0 [-0.5, 6.6] | 0.297 | -0.1 [-1.4, 2.3] | 1.000 |
| **r[Hb]** | -1.7 [-5.2, 1.8] | 0.167 | -0.3 [-1.9, 2.1] | 0.737 | +0.1 [-0.9, 1.5] | 0.652 | +1.6 [-0.1, 2.7] | 0.156 | -0.3 [-0.8, 0.8] | 1.000 |
| **(Δ, mmHg)** | **Effect Size** | **p** | **Effect Size** | **p** | **Effect Size** | **p** | **Effect Size** | **p** | **Effect Size** | **p** |
| **SBP** | +35.7 [22.5, 90.5] | <0.001* | +37.7 [20.9, 52.3] | <0.001* | +50.0 [22.2, 58.1] | 0.008* | +28.9 [11.7, 54.3] | 0.031 | +10.8 [5.6, 20.4] | 0.125 |
| **DBP** | +13.9 [10.1, 21.1] | <0.001* | +11.8 [8.7, 15.4] | <0.001* | +8.6 [4.9, 11.9] | 0.004* | +7.2 [3.4, 9.7] | 0.016* | +3.9 [0.7, 6.8] | 0.125 |
| **CPP** | +13.7 [9.0, 20.2] | <0.001* | +11.5 [8.7, 14.4] | <0.001* | +9.6 [4.5, 11.8] | 0.016 | +7.6 [2.4, 9.9] | 0.031 | +1.1 [0.6, 5.3] | 0.250 |
| Effect Size reported as median [interquartile range].  *Adjusted p<0.05 after false discovery rate correction for repeated comparisons.  Abbreviations: Δ, change in value; n, sample size; rCBF, relative cerebral blood flow; rPbtO_2_, relative partial pressure of oxygen in brain tissue; rStO_2_, relative cerebral tissue oxygen saturation; rTHC, relative total hemoglobin concentration; r[HbO_2_], relative concentration of oxy-hemoglobin; r[Hb], relative concentration of deoxy-hemoglobin; SBP, systolic aortic blood pressure; DBP, diastolic aortic blood pressure; CPP, coronary perfusion pressure. | | | | | | | | | | |
