# Supplementary material for: Epinephrine’s effects on cerebrovascular and systemic hemodynamics during cardiopulmonary resuscitation
Source: Crit Care. 2020 Sep 29;24:583. doi: 10.1186/s13054-020-03297-4 (PMC7522922; doi:10.1186/s13054-020-03297-4)
Supplement: Supplementary file 4 — Additional file 4. [file 13054_2020_3297_MOESM4_ESM.docx]

| **Table S3: Change in Dose Efficacy with Repeated Doses of Intra-Arrest Epinephrine, Comparison to Dose 1** | | | | | | | | |
| --- | --- | --- | --- | --- | --- | --- | --- | --- |
|  | **Dose 2 (n=20)** |  | **Dose 3 (n=9)** |  | **Dose 4 (n=7)** |  | **Dose 5 (n=4)** |  |
| **(Δ, % Baseline)** | **Δ Effect Size** | **p** | **Δ Effect Size** | **p** | **Δ Effect Size** | **p** | **Δ Effect Size** | **p** |
| **Invasive rCBF** | -4.1 [-16.0, 2.2] | 0.079 | -3.2 [-16.9, 3.0] | 0.469 | -5.2 [-13.0, -2.0] | 0.312 | -12.0 [-23.1, 4.9] | 0.500 |
| **Non-Invasive rCBF** | -3.9 [-13.6, 1.1] | 0.053 | -1.6 [-5.4, -1.0] | 0.297 | -4.4 [-9.4, -1.7] | 0.219 | -3.4 [-7.7, 3.7] | 0.625 |
| **rPbtO_2_** | -2.3 [-7.4, 1.1] | 0.015 | -3.0 [-6.7, 1.5] | 0.250 | -2.7 [-5.9, 1.4] | 0.375 | -1.9 [-6.3, 0.4] | 0.625 |
| **rStO_2_** | -3.3 [-12.7, 0.2] | 0.014* | -6.2 [-9.6, -2.4] | 0.004* | -5.3 [-6.7, -4.5] | 0.016* | -4.4 [-7.2, -3.5] | 0.125 |
| **rTHC** | -6.6 [-26.8, 0.3] | 0.064 | -4.1 [-31.4, 2.3] | 0.301 | -4.1 [-7.3, 1.1] | 0.469 | -4.4 [-5.7, -1.0] | 0.250 |
| **r[HbO_2_]** | -3.2 [-18.7, -0.7] | 0.006* | -8.7 [-14.2, -3.8] | 0.004* | -7.5 [-10.5, -4.1] | 0.016* | -4.6 [-10.3, -4.2] | 0.125 |
| **r[Hb]** | +0.3 [-1.5, 5.2] | 0.370 | +0.9 [-1.9, 8.7] | 0.359 | +1.4 [-1.2, 7.0] | 0.297 | +1.2 [-2.2, 4.8] | 0.625 |
| **(Δ, mmHg)** | **Δ Effect Size** | **p** | **Δ Effect Size** | **p** | **Δ Effect Size** | **p** | **Δ Effect Size** | **p** |
| **SBP** | -6.4 [-29.2, 2.1] | 0.062 | +9.1 [-20.0, 23.4] | 0.910 | +9.4 [-14.6, 23.5] | 0.688 | -15.6 [-51.8, 4.3] | 0.375 |
| **DBP** | -0.8 [-7.2, 1.6] | 0.135 | -7.5 [-11.2, 0.3] | 0.250 | -4.9 [-9.3, 2.7] | 0.375 | -6.2 [-15.9, 1.0] | 0.375 |
| **CPP** | -0.8 [-6.9, 2.9] | 0.227 | -6.5 [-10.3, 4.2] | 0.461 | -5.1 [-9.5, 5.3] | 0.562 | -8.2 [-17.1, 1.6] | 0.500 |
| Effect Size reported as median [interquartile range].  *Adjusted p<0.05 after false discovery rate correction for repeated comparisons.  Abbreviations: Δ, change in value; n, sample size; rCBF, relative cerebral blood flow; rPbtO_2_, relative partial pressure of oxygen in brain tissue; rStO_2_, relative cerebral tissue oxygen saturation; rTHC, relative total hemoglobin concentration; r[HbO_2_], relative concentration of oxy-hemoglobin; r[Hb], relative concentration of deoxy-hemoglobin; SBP, systolic aortic blood pressure; DBP, diastolic aortic blood pressure; CPP, coronary perfusion pressure. | | | | | | | | |
